# The effects of zooprophylaxis and other mosquito control measures against malaria in Nouna, Burkina Faso

**DOI:** 10.1186/1475-2875-8-283

**Published:** 2009-12-09

**Authors:** Shelby S Yamamoto, Valérie R Louis, Ali Sié, Rainer Sauerborn

**Affiliations:** 1 Institute of Public Health, University of Heidelberg, Im Neuenheimer Feld 324, 69120 Heidelberg, Germany; 2 Centre de Recherche en Santé de Nouna, BP 02, Nouna, Burkina Faso

## Abstract

**Background:**

In the absence of large scale, organized vector control programmes, individual protective measures against mosquitoes are essential for reducing the transmission of diseases like malaria. Knowledge of the types and effectiveness of mosquito control methods used by households can aid in the development and promotion of preventive measures.

**Methods:**

A matched, population-based case control study was carried out in the semi-urban region of Nouna, Burkina Faso. Surveys and mosquito captures were conducted for each participating household. Data were analysed using conditional logistic regression and Pearson's product-moment correlations.

**Results:**

In Nouna, Burkina Faso, the main types of reported mosquito control measures used included sleeping under bed nets (insecticide-treated and untreated) and burning mosquito coils. Most of the study households kept animals within the compound or house at night. Insecticide house sprays, donkeys, rabbits and pigs were significantly associated with a reduced risk of malaria only in univariate analyses.

**Conclusion:**

Given the conflicting results of the effects of zooprophylaxis from previous studies, other community-based preventive measures, such as bed nets, coils and insecticide house-spraying, may be of more benefit.

## Background

Africa is particularly vulnerable to malaria for several reasons, including being exposed to the most severe form of the disease, having inadequate resources to bear the economic burden of the consequences and having to cope with the lack of proper infrastructure to effectively treat cases [1]. Hence, prevention of the disease becomes paramount. The use of individual methods of protection are particularly important, especially in areas lacking any formal mosquito control programmes, like Burkina Faso. Bed nets, window screens, house sprays, ceilings, closed eaves and in some cases, zooprophylaxis can reduce the risk of malaria [2-6]. The evaluation of the different types of mosquito control methods used by households may be used to aid in the development and promotion of preventive measures that can be more readily integrated into communities. Thus, the aim of this study was to assess the reported use of different mosquito control methods among residents and their effects on the risk of malaria in the semi-urban area of Nouna, Burkina Faso.

## Methods

### Study site

The study site of Nouna, Burkina Faso is located in the Kossi province, approximately 300 km from the capital city of Ouagadougou. Average annual rainfall in the region is about 700 mm. The area is holoendemic for *Plasmodium falciparum *malaria although the peak transmission season is from June to October, during the wet season. Malaria vectors in the region include *Anopheles gambiae*, *Anopheles funestus *and *Anopheles arabiensis*. However, the predominant malaria vector is *An. gambiae*.

### Study design and population

A population-based, 1:2 matched case-control study was carried out with 117 cases and 221 controls. Cases included women (15-45 years) and children (≤ 9 years) recruited from those who were tested for malaria at the Centre de Recherche en Santé de Nouna Laboratory. The malaria risks posed to children are high in this region with the incidence estimated as 0.21 per child per month [7]. In addition, women of child-bearing age were included as they are often the primary caretakers of families and thus their health can affect entire households. Uncomplicated malaria cases were confirmed by thin and thick blood smears. Cases were matched to controls who did not visit the laboratory for malaria testing during the period of recruitment. Controls were selected from an extensive Demographic Surveillance System (DSS) database, which includes recent demographic and health data from 74,000 residents in Nouna and the surrounding areas [8]. Matching criteria included age (± 3 years), sex, ethnicity and residence (town sector).

### Data collection and analysis

Respondents (women and caretakers of the recruited children) were interviewed about different household protective measures against mosquitoes during the wet season. Mosquito captures were also performed over the course of one night for each household. Battery-powered light traps (CDC) with incandescent bulbs were used to capture mosquitoes in the sleeping rooms of the cases and controls. The trap was suspended above the floor and mounted close to the bed of the case or control but outside any mosquito nets. Pyrethrum spray captures were also performed the following morning. White drop cloths were laid down on the floors and a locally available insecticide was applied to the walls, furniture and roof for approximately one minute after closing the doors and windows. The participants were instructed to follow their usual daily mosquito control routines.

The responses were analyzed with SAS 9.1 (SAS Institute Inc., Cary, NC, USA) using univariate and multivariate conditional logistic regression. Those variables significant in univariate analyses at the *P *= 0.20 level were subsequently modeled by multivariate analysis. Multivariate models were adjusted for reported bed net use (yes/no) and level of education (none, primary, secondary or higher).

## Results

The demographic characteristics of the cases and controls were similar (e.g. religion, household size, relationship of the respondent to the household head). An exception was the highest reported level of education. More cases than controls reported having secondary education or higher (OR = 2.35, 95%CI: 1.18-4.70), therefore this variable was included as a potential confounder in multivariate models.

Most cases (99.2%) and controls (98.6%) reported using at least one mosquito preventive measure during the wet season. These included mainly sleeping under bed nets (both insecticide-treated and untreated) and burning mosquito coils (Table 1). Few used smoke from burned plant material regularly (≥ once per week) as a means to repel mosquitoes. Of those households that did, plants were burned mostly in the bedroom (11.2%) and the living areas (6.5%).

**Table 1:** Frequency of different reported mosquito control methods used in Nouna during the rainy season

Type of mosquito control	Cases (%)	Controls* **(%)**
Untreated bed net**	61 (52.1)	112 (50.7)
Insecticide-treated bed net**	64 (54.7)	109 (49.3)
Mosquito coils	79 (67.5)	142 (64.3)
Insecticide house sprays	16 (13.6)	14 (6.3)
Smoke from plants (≥ once per week)	13 (11.1)	32 (14.5)

* Pooled controls (control 1 and control 2)

** Some case and control households reported using both insecticide-treated and untreated nets

Several different types of animals were also owned or kept by the households. Animals were frequently kept within household compounds by both the cases (89.6%) and controls (87.2%) at night. Of the different types of livestock, cattle were most often found within the compound (Table 2). Donkeys were found to be significantly and positively correlated with female *An. gambiae *abundance in sleeping rooms (Pearson's r = 0.21, *P *= 0.0002). Separate analyses by unfed (Pearson's r = 0.16, *P *= 0.0045), fed (Pearson's r = 0.22, *P *< 0.0001) and sub-gravid (Pearson's r = 0.16, *P *= 0.02) status also produced significant correlations. No associations were found with any other types of animals.

**Table 2:** Types of animals kept within compounds or houses at night

Animals kept in compound	Cases (%)	Controls* **(%)**
Poultry	60 (51.3)	108 (48.9)
Cows	56 (47.9)	114 (51.6)
Sheep	49 (41.9)	101 (45.7)
Donkeys	29 (24.8)	77 (34.8)
Goats	26 (22.2)	47 (21.3)
Cats	26 (22.2)	43 (19.5)
Dogs	25 (21.4)	36 (16.3)
Others (e.g. rabbits)	11 (9.4)	36 (16.3)
Pigs	3 (2.6)	20 (9.1)
Horses	0 (0.0)	1 (0.5)

* Pooled controls (control 1 and control 2)

Reported use of insecticide-treated and untreated bed nets as well as mosquito coils did not significantly affect the risk of malaria in univariate analyses (Table 3). Conversely, when animals such as donkeys, rabbits and pigs were kept within compounds, a statistically significant protective effect was observed. An effect was not found with other animals such as cows, sheep, goats and poultry. Nonetheless, no variables included in multivariate models remained significant (*P *< 0.05), after adjusting for reported bed net use and level of education.

**Table 3:** Univariate analysis of the effects of different mosquito control practices on malaria risk

Description	OR	95% CI	*P*-value
*Type of mosquito control*			
Untreated bed net	1.04	0.66-1.63	0.88
Insecticide-treated bed net	1.23	0.79-1.93	0.36
Mosquito coils	1.24	0.73-2.00	0.47
**Insecticide house sprays**	**2.49**	**1.11-5.59**	**0.03**
Smoke from plants	0.74	0.35-1.56	0.43
			
*Animals kept in compound*			
Poultry	1.14	0.68-1.90	0.63
Cows	0.84	0.45-1.54	0.57
Sheep	0.84	0.51-1.37	0.48
**Donkeys**	**0.59**	**0.34-1.01**	**0.05**
Goats	1.08	0.60-1.93	0.80
Cats	1.17	0.66-2.08	0.59
Dogs	1.41	0.73-2.73	0.31
**Others (i.e. rabbits)**	**0.52**	**0.25-1.09**	**0.09**
**Pigs**	**0.26**	**0.07-0.89**	**0.03**

OR, odds ratio

95% CI, 95% confidence interval

Bolded values are those significant at the *P *< 0.20 level

## Discussion

Most of the respondents reported using some protective measures against mosquitoes during the wet season, a finding also echoed by Samuelsen *et al *[9]. Sleeping under bed nets and burning mosquito coils were the most popular forms of control during the rainy season. In fact, reported bed net use was very high. This could have been the result of promotions in the region encouraging the use of insecticide-treated bed nets [3]. However, actual bed net use was not investigated in this study and could have been over-reported by the respondents, particularly since knowledge of the protective effects of bed nets does not necessarily translate into actual net use [10]. Insecticide house sprays were not a popular method of mosquito control in Nouna, likely because of the costs associated with such measures (Ouédraogo pers com. 2008). Similarly, smoke from the burning of plant material was also not as extensively used as in other regions like The Gambia [11].

The evidence regarding the effectiveness of zooprophylaxis is generally not clear. Keeping animals near houses can shift the biting behaviour of mosquitoes away from people and, therefore, mitigate malaria risk [12]. Conversely, sharing the house with livestock could also increase the risk of malaria. Greater numbers of mosquitoes may be initially attracted to households because of the presence of animals but then end up preferentially feeding on humans [13,14]. In The Gambia, it was found that animals conferred little or no protection against *An*. *gambiae *and that the presence of cattle did not result in greater numbers of zoophilic species or a change in species composition. Moreover, the human blood index (HBI), or proportion of human-fed mosquitoes, was not altered among indoor resting mosquitoes between households with and without cattle [15].

In this study, most domestic animals sleeping within the compound or house were not associated with either an increased or decreased risk of malaria. Donkeys, rabbits and pigs, however, were associated with some zooprophylactic effect in univariate analyses. One possible explanation for the protective effect observed in this study could be that if vector breeding sites are closer to animal enclosures than to houses, mosquitoes may choose to feed on livestock as opposed to humans [16,17]. We observed significant, positive correlations between donkeys and the abundance of female *An. gambiae *mosquitoes captured in the sleeping rooms of the participants. This could suggest that donkeys, in particular, attract mosquitoes. Likewise, Pålsson *et al *[18] found that pigs, but not goats, were associated with increased numbers of mosquitoes collected from bedrooms in Guinea Bissau. Nonetheless, when bed net use or other behaviours, such as staying indoors are considered, animals may be a more accessible blood meal source [19,20]. It is also possible that certain types of animals, in particular equines such as horses and donkeys, exert stronger zooprophylactic effects. Bøgh *et al *[21] observed that the preference of *An*. *gambiae *for horses was strong in The Gambia, although since horse ownership was also very high, accessibility may have also influenced host choice. Another study in The Gambia also found that horses located near houses were correlated with lower numbers of mosquitoes in rooms, whereas no significant association was found with cattle [22]. Additional evidence suggests that the *An*. *gambiae *mosquitoes found in certain areas of West Africa may be less anthropophilic compared to those found in other areas, which may also explain some of the zooprophylactic effects observed in the study [23,24].

Zooprophylaxis may lower the risk of malaria in this region. However, before this method of mosquito control could be recommended, other factors need be considered such as the potential for increased mosquito survival from greater feeding success or the creation of additional vector breeding sites from hoof prints, animal enclosures and water tanks [25,26]. It would also be necessary to further investigate separately the impact, in particular, of donkey, rabbit and pig proximity on malaria outcome and acquire more knowledge about mosquito anthropophilic biting behaviour in the area.

A study limitation was that mosquito captures were only conducted for one night. As a result, there was no way to determine if the species composition and counts obtained were representative of the conditions typically found in households. Nonetheless, cases and controls were largely sampled on the same day to avoid confounding from weather conditions. Also, sampling was not carried out on public holidays or other days that might have affected collections. Therefore, cases and controls were assumed to be equally affected by environmental factors and any bias non-differential. An additional limitation of this study was that active testing for malaria among the controls did not occur before recruitment, which may have biased the results towards the null. However, no controls reported having malaria during the recruitment period and none from the second field season had a fever (≥37.5°C) at the time of data collection.

## Conclusion

Given the infrequent use of smoke from the burning of plants and likely respiratory side effects as well as the interesting but unconfirmed results of zooprophylaxis, other measures to protect against mosquitoes may be of greater benefit in this region. Bed nets and mosquito coils have already gained wide acceptance and should be further encouraged. Community-based house spraying programmes may also be an effective tool.

## Competing interests

The authors declare that they have no competing interests.

## Authors' contributions

SY, AS, RS were responsible for designing the study, data collection was conducted by SY and AS, SY and VRL examined, analysed and interpreted the data, SY, VRL and RS drafted the paper. All authors read, edited and approved the final manuscript.
